# Association Between Open Payments–Reported Industry Transfers of Value and Prostaglandin Analog Prescribing in the US

**DOI:** 10.1001/jamaophthalmol.2022.2757

**Published:** 2022-07-28

**Authors:** Andrew M. Nguyen, Kelly E. Anderson, Gerard Anderson, Thomas V. Johnson

**Affiliations:** 1 Johns Hopkins University School of Medicine, Baltimore, Maryland; 2 University of Colorado Skaggs School of Pharmacy and Pharmaceutical Sciences, Aurora; 3 Johns Hopkins Bloomberg School of Public Health, Baltimore, Maryland; 4 Glaucoma Center of Excellence, Wilmer Eye Institute, Johns Hopkins University School of Medicine, Baltimore, Maryland

## Abstract

**Importance:**

Reported transfers of value (TOV) from pharmaceutical companies have been associated with greater use of branded anti–vascular endothelial growth factor agents by ophthalmologists, but payment under the Medicare Part B buy-and-bill model includes a financial incentive to choose costlier agents, potentially confounding analyses of pharmaceutical TOV and prescribing patterns. How these reported TOV are associated with prescribing patterns for prescription eye drops, not subject to the incentives created by Part B payments, should be considered.

**Objective:**

To assess the association between prostaglandin analog (PGA) eye drop prescribing and reported nonresearch TOV by makers of branded PGAs to US vision care professionals.

**Design, Setting, and Participants:**

This retrospective cohort analysis used a 20% nationally representative sample of 2018 Medicare Part D claims and industry TOV reported to the Open Payments program.

Optometrists and ophthalmologists who had more than 10 claims for PGA drops in the 20% sample were analyzed. Analysis took place from June 2021 to February 2022.

**Main Outcomes and Measures:**

Multivariable logistic regression assessing the association between membership in strata of reported TOV and branded PGA prescribing rate, controlling for prescriber demographic factors, local area practices, total PGA prescribing volume, and plan formularies involved.

**Results:**

A total of 20 612 ophthalmologists and 5426 optometrists (7449 [29%] female and 18 589 [71%] male) prescribed PGA eye drops. Of these, 9685 (37%) were reported to have received TOV from manufacturers of branded PGAs in 2018, totaling $5 060 346. The median (IQR) reported TOV was $65 ($24-$147). Multivariable logistic regression showed that the predicted probability of primarily prescribing branded PGAs among prescribers who reported receiving no TOV was 12.9% (95% CI, 12.4%-13.4%). This figure increased to 19.6% (95% CI, 18.8%-20.4%) among prescribers receiving TOV, a 50% increase. There was a dose-response association, such that the top 10% of TOV recipients had a 29.2% probability (95% CI, 26.4%-31.9%) of preferential branded use.

**Conclusions and Relevance:**

While the median reported TOV to a PGA prescriber was relatively low in this study, there was a positive association between amount of reported nonresearch TOV received from PGA makers and the frequency of branded PGA use. This shows that small reported TOV were associated with differences in prescribing. High rates of branded PGA prescribing may pose a cost burden to patients that affects adherence. Clinicians and policy makers should be aware of these associations.

## Key Points

**Question:**

Are transfers of value (TOV) reported by makers of branded prostaglandin analog (PGA) eye drops associated with a greater likelihood of prescribing branded PGAs?

**Findings:**

In this cohort study, recipients of TOV were more likely to prescribe branded PGAs compared to prescribers with no reported TOV, even with a relatively low TOV median. A dose-response association was observed, which was robust even when excluding prescribers with the greatest TOV.

**Meaning:**

The findings in this study show that even small TOV from pharmaceutical companies were associated with greater prescription of branded drugs.

## Teaser

This cohort study evaluates the association of prostaglandin analog eye drop prescribing and transfers of value reported by makers of branded prostaglandin analogs to US vision care professionals.

## Introduction

Established in 2010 by the Physician Payments Sunshine Act under the Affordable Care Act, the Open Payments program requires manufacturers of drugs, medical devices, and medical supplies to report to the Centers for Medicare & Medicaid Services (CMS) their payments and transfers of value (TOV) (including meals, travel fees, speaking fees, and gifts, discrete from research support) to physicians and teaching hospitals.^1^ Since the database was made publicly available in 2014, there have been several efforts to examine the impact of reported industry TOV.^2,3,4,5,6,7,8^ Systematic reviews across a range of specialties consistently identify a positive association between reported nonresearch TOV and greater prescribing of drugs of interest.^9,10^

Many physicians interact with and have positive perceptions of pharmaceutical sales representatives, and from them, physicians receive TOV and information about branded drugs and their use.^11^ Information provided without TOV, as in industry-sponsored continuing medical education events, has also been found to be associated with higher branded drug use.^12^

Within the ophthalmology literature, assessments of the association between reported industry TOV and prescribing habits have focused on anti–vascular endothelial growth factor agents, delivered by intravitreal injection for the management of proliferative diabetic retinopathy and neovascular age-related macular degeneration.^13,14,15,16^ However, there is a potential confounding factor in these analyses, as ophthalmologists administering anti–vascular endothelial growth factor agents are paid by Medicare under Part B buy and bill based on the average sales price of the drug plus a percentage add-on payment. Coupled with the potential effect of industry TOV on prescribing habits, ophthalmologists may then be subject to dual incentives to select a more costly anti–vascular endothelial growth factor agent, complicating the analysis of whether industry TOV are independently associated with greater use of a drug.^17^

Prescription eye drops are used by ophthalmologists and optometrists for the management of ocular disease and are covered under Medicare Part D, which does not offer financial incentives for prescribing more expensive agents. This provides an opportunity to isolate and investigate the effect of reported industry TOV. Prostaglandin analogs (PGAs), used in the management of glaucoma, are particularly amenable to such investigation, as generic latanoprost exists as an alternative to higher-cost bimatoprost (Lumigan [Allergan]), travoprost (Travatan Z [Novartis]), and tafluprost (Zioptan [Merck]), which are not consistently superior in reducing intraocular pressure.^18,19^ Additionally, glaucoma is a chronic condition, so higher-cost branded PGAs can impose a long-term financial burden on patients that could impact treatment adherence.^20,21^ Indeed, studies have shown that patients who started taking the lower-cost drug, latanoprost, exhibit greater odds of treatment adherence and lower odds of drug switching than patients prescribed other PGAs.^22^

This study characterizes the association between reported receipt of nonresearch TOV from makers of branded PGAs and PGA prescribing patterns for vision care professionals in 2018 using CMS Part D claims, focusing on the specific impact of small TOV, as there is a common perception among physicians^23,24,25^ and patients^26^ that small gifts such as meals are unlikely to influence prescribing behavior.

## Methods

### Study Design and Participants

We performed a retrospective cohort analysis of PGA eye drop prescribers in 2018 using a nationally representative 20% sample of Medicare beneficiaries in the 2018 CMS Part D Event data set.^27^ This data set contained 2 545 761 claims for latanoprost, Xalatan, travoprost, Travatan, bimatoprost, Lumigan, tafluprost, and Zioptan in drop form. Findings are reported in accordance with Strengthening the Reporting of Observational Studies in Epidemiology (STROBE) reporting guideline. This study was defined to be exempt from institutional review board review by the Johns Hopkins Bloomberg School of Public Health Institutional Review Board because the data set provided by CMS were deidentified through randomized beneficiary identification numbers and contained only zip codes for beneficiary-level data.

The number of unique Medicare beneficiaries, total number of claims, days supplied of each drug, and unique plan formularies were tabulated for each prescriber using their National Provider Identification (NPI) number. To eliminate data anomalies, claims belonging to a beneficiary with more than 455 days (1 year + 90 days) of a single PGA prescribed by a single clinician in 2018 were dropped. To ensure that clinicians had sufficient observations from which to draw conclusions, those with fewer than 10 claims in the 20% sample (corresponding to fewer than 50 claims for Medicare beneficiaries in 2018) were also excluded. Our analytic sample included 35 927 unique prescribers.

### Procedures

As Open Payments does not document NPI, reported industry TOV information was merged from Open Payments to CMS Part D claims data using the 2017-2018 Dollars for Docs NPI-to-Open Payments Crosswalk file created by ProPublica,^28^ which matches NPI to 99% of prescribers in Open Payments using National Plan & Provider Enumeration System data from 2011, 2015, 2016, and 2018.^29^ Additional demographic information at the prescriber level was merged from National Plan & Provider Enumeration System^30^ data using NPI and from Rural Urban Commuting Area designations^31^ using zip codes (eFigure 1 in the Supplement).

Prescriber specialty and subspecialty are not required fields in Part D Event files and Open Payments submissions, so this information was supplemented using 2017-2018 CMS Outpatient Revenue Center Claims^32^ and Carrier Claims.^33^ Where possible, NPI numbers were labeled as belonging to an optometrist, nonglaucoma specialist ophthalmologist, or glaucoma specialist based on specialty codes listed on claims and in the National Plan & Provider Enumeration System. In addition, we discriminated ophthalmologists and optometrists based on the ability of only ophthalmologists to bill for 1 or more of the following procedures in a year: tube shunt placement or trabeculectomies, cataract surgery, keratoplasties, vitrectomies, and intravitreous injections. Glaucoma specialists were identified as those who billed for 3 or more tube shunts and/or trabeculectomies in a year (eFigure 2 in the Supplement).

It was possible to match 34 931 of 35 855 PGA eye drop prescribers with an identified specialty (97%) between Open Payments, Part D Event claims, and 2017-2018 Outpatient and Carrier Claims. A total of 26 070 prescribers were identified as vision care professionals (optometrists or ophthalmologists), and 26 038 were retained for analysis after dropping those with missing sex and geographical location data.

For each prescriber, the total value of reported nonresearch TOV from makers of branded PGAs (Merck, Allergan, and Novartis) was aggregated by NPI. Branded PGA use for each prescriber was calculated as a percentage of total days supplied of PGA eye drops. Previous works comparing Open Payments data and individual-level prescribing patterns identified prescriber sex,^3,5,13,34,35,36,37^ specialty,^2,3,4,5,6,7,8,34,35,36,37,38,39,40,41,42^ total prescribing volume,^5,34,35,39,41^ US Census region,^34,35^ and zip code–based urban vs rural designation^5^ as potentially significant covariates. Our analysis showed collinearity between US Census region and USPS Rural Urban Commuting Area urban vs rural designation, with Rural Urban Commuting Area being a stronger predictor of both branded PGA use and reported TOV from PGA makers. Accordingly, our model used prescriber sex, specialty (optometry vs ophthalmology), glaucoma subspecialty, log-transformed total PGA prescribing volume, or urban location as covariates. The simple correlation between variables is shown in eTable 1 in the Supplement.

There is evidence that physicians conform to prevailing practice patterns in their geographic region.^43^ The effect of peer preferences was controlled for by using the mean rate of branded PGA use in each 3-digit zip code as a covariate. Plan formularies in each prescribers’ patient panel may limit their choice of therapeutic agents, so each prescriber’s number of unique plan formularies seen was also included as a covariate to capture the degree of limitation.

### Outcomes and Statistical Analysis

Statistical analyses were performed using Stata version 16 (StataCorp) with the package *dunntest*. Descriptive statistics, Mann-Whitney *U* tests, and Kruskal-Wallis *H* tests with Bonferroni corrections were used to characterize prescribing patterns and reported TOV patterns across prescriber-level characteristics. Multiple multivariable logistic regressions were used to assess the association between membership in each stratum of reported TOV and high branded PGA use, controlling for other prescriber characteristics, to investigate the hypothesis that there is some threshold value of TOV below which there would be no association with prescribing. Sensitivity analyses included subgroup models for optometrists and ophthalmologists separately and separating TOV by type (food and drink, travel, speaking fees, consulting, etc). Results are reported as marginal probabilities for the variable of interest, holding other covariates at their means. *P* values were 2-sided and adjusted for multiple comparisons by Bonferroni correction. *P* values were statistically significant at .05. Analysis took place between July 2021 and February 2022.

## Results

Of 26 038 vision care professionals who prescribed PGA eye drops, 7449 (29%) were female and 18 589 (71%) were male; 21 438 (82%) practiced in an urban location. The set contained 5426 optometrists (21%) and 20 612 ophthalmologists (79%), including 1103 glaucoma specialists. Formulary constraints varied, with 1025 (4%) having 1 unique plan formulary in their panel, 9228 (35%) having 2 to 5, 7763 (30%) having 6 to 10, and 8022 (31%) having greater than 10. The median (IQR) total days of PGAs supplied per prescriber was 1778 (831-4319). The median (IQR) branded PGA use rate at the 3-digit zip code level was 26% (20.4%-30.6%). Overall, 16 353 prescribers (63%) were not reported to have received TOV from makers of branded PGAs in 2018 (Table).

**Table.** Demographic Characteristics of Prescribers of Prostaglandin Analogs (PGAs) in 2018

Characteristic	No. (%)					
	All prescribers	Prescribers who did not receive TOV	Prescribers who received TOV	TOV recipients		
				Top 50%^a^	Top 25%^b^	Top 10%^c^
Overall, No.	26 038	16 353	9685	4843	2422	968
Sex						
Female	7449 (29)	4815 (29)	2634 (27)	1332 (28)	663 (27)	226 (23)
Male	18 589 (71)	11 538 (71)	7051 (73)	3511 (72)	1759 (73)	746 (77)
Location						
Not urban	4600 (18)	3773 (23)	827 (9)	318 (7)	96 (4)	26 (3)
Urban	21 438 (82)	12 580 (77)	8858 (91)	4525 (93)	2326 (96)	942 (97)
Specialty						
Optometry	5426 (21)	3769 (23)	1657 (17)	751 (16)	384 (16)	111 (11)
Ophthalmology	20 612 (79)	12 584 (77)	8028 (83)	4092 (84)	2074 (86)	857 (89)
Subspecialty						
Not glaucoma	24 935 (96)	15 989 (78)	8946 (92)	4325 (89)	2066 (85)	761 (79)
Glaucoma	1103 (4)	364 (2)	739 (8)	518 (11)	356 (15)	207 (21)
No. of plan formularies						
1	1025 (4)	904 (6)	121 (1)	57 (1)	16 (1)	4 (0.4)
2-5	9228 (35)	7050 (43)	2178 (22)	1079 (22)	459 (19)	161 (17)
6-10	7763 (30)	4896 (30)	2867 (30)	1368 (28)	619 (26)	225 (23)
>10	8022 (31)	3503 (21)	4519 (47)	2339 (49)	1328 (54)	578 (60)
Total days supplied of PGAs, median (IQR)	1778 (831-4319)	1385 (721-3105)	3022 (1214-6487)	3162 (1207-7384)	3984 (1398-9241)	4811 (1566-10795)
3-Digit zip code–branded PGA use, median (IQR), %	26.0 (20.4-30.6)	25.3 (19.7-29.7)	27.4 (22.2-31.4)	27.6 (22.8-31.5)	28.3 (24.0-32.1)	28.5 (24.6-32.1)
Total reported TOV, $	5 060 346	0	5 060 346	4 920 495	4 661 511	4 363 829

Abbreviation: TOV, transfer of value.

^a^ Reported receipt of >$65 from branded PGA makers in 2018.

^b^ Reported receipt of >$147 from branded PGA makers in 2018.

^c^ Reported receipt of >$288 from branded PGA makers in 2018.

**Figure 1.**
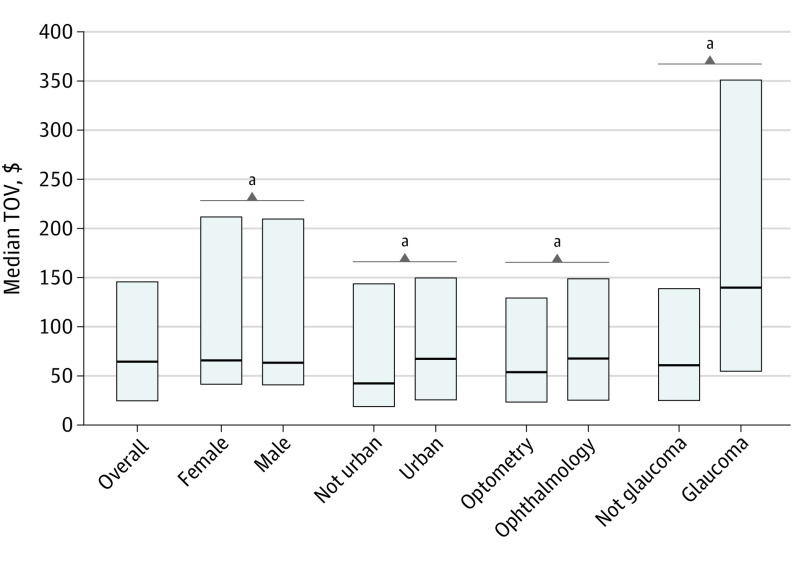
Reported Transfers of Value (TOV) From Prostaglandin Analog Makers Plots show median (IQR) reported TOV for prescribers with reported receipt of TOV in each category. Significance calculated by Wilcoxon rank sum test using Bonferroni correction. ^a^*P* ≤ .001.

The median (IQR) total value of reported nonresearch TOV received from PGA makers was only $65 ($24-$147). Urban prescribers received more than rural prescribers (median [IQR], $68 [$25-$151] vs $43 [$17-$102]; *P* < .001). Ophthalmologists received more than optometrists (median [IQR], $68 [$25-$150] vs $54 [$23-$131]; *P* < .001), and glaucoma specialists received more than nonglaucoma specialists (median [IQR], $140 [$54-$352] vs $61 [$24-$140]; *P* < .001) (Figure 1). The distribution of reported TOV was skewed, with the top 25% of recipients receiving 92% of the total value of reported TOV from PGA makers ($4 661 511 of $5 060 346) and the top 10% receiving 86% ($4 363 829 of $5 060 346) (Table). TOV descriptive statistics by company are documented in eTable 2 in the Supplement.

**Figure 2.**
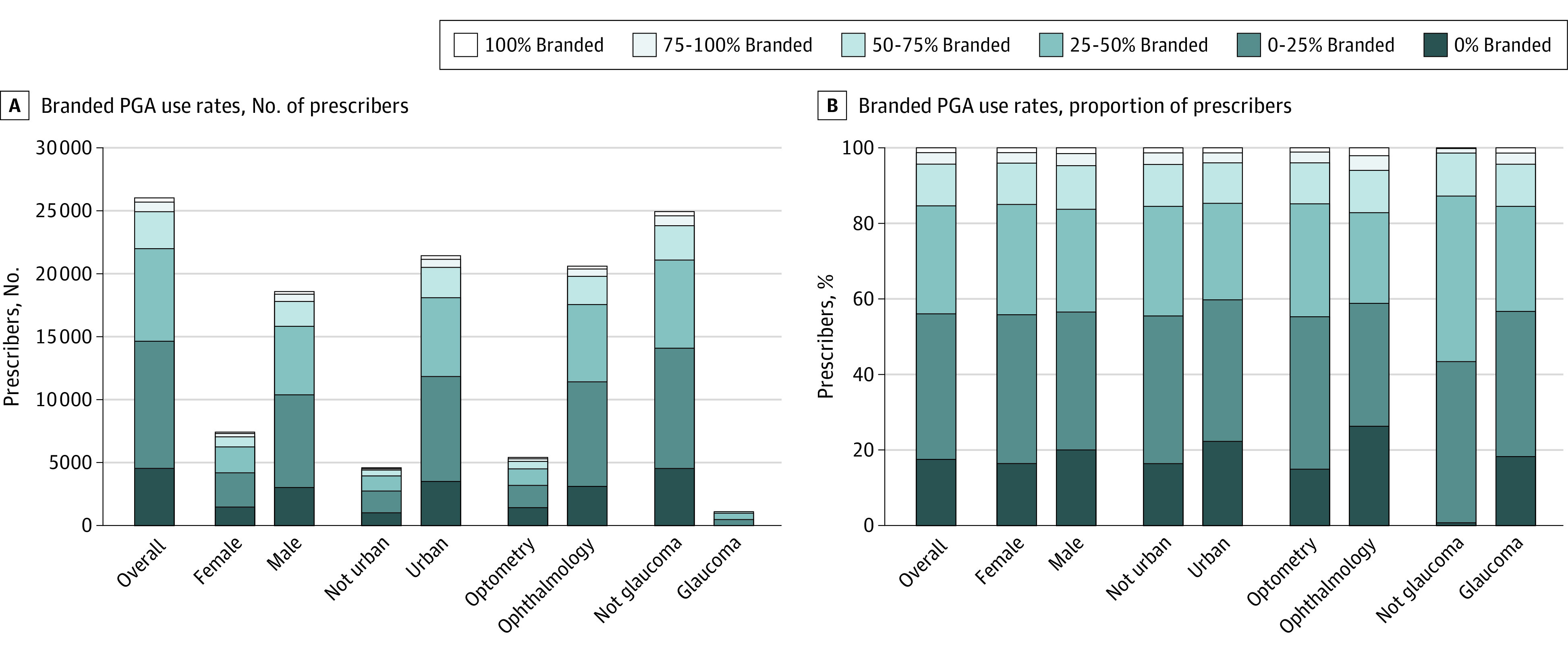
Branded Prostaglandin Analog (PGA) Use A, The absolute number of prescribers in each category for each level of branded PGA use, calculated as total days supplied of branded PGAs out of total days supplied of any PGA. B, The proportion of prescribers in each category for each level of branded PGA use.

There was substantial variation across prescribers in percentage of branded PGA use. Overall, 4559 prescribers (18%) did not prescribe any branded PGAs, while 17 480 (67%) prescribed branded PGAs but less than half of the time. Overall, there were 2893 prescribers (11%) who branded PGAs between 50% and 75% of the time, and 1106 (4%) prescribed branded PGAs more than 75% of the time (Figure 2).

Multivariable logistic regression, controlling for prescriber characteristics and local prescribing patterns, showed that reported receipt of any nonresearch TOV was associated with a greater likelihood of being a high prescriber of branded PGAs. A high prescriber was defined as prescribing branded PGAs more than 50% of the time by days supplied. The predicted probability, holding other variables at their means, of being a high prescriber with no reported TOV was 12.9% (95% CI, 12.4%-13.4%; *P* < .001) compared with 19.6% (95% CI, 18.8%-20.4%; *P* < .001) with reported receipt of any TOV. Prescribers in the top 50% of TOV recipients had a 1.8-fold greater predicted probability of being a high prescriber (22.5% [95% CI, 21.3%-23.7%]; *P* < .001), and prescribers in the top 25% of TOV recipients had a 2-fold greater predicted probability (26.3% [95% CI, 24.6%-28.0%]; *P* < .001). The top 10% had a 2.3-fold greater predicted probability (29.2% [95% CI, 26.4%-31.9%]; *P* < .001). This shows a dose-response association, where membership in higher strata of reported TOV receipt was associated with an increasingly greater probability of being a high prescriber of branded PGA eye drops.

**Figure 3.**
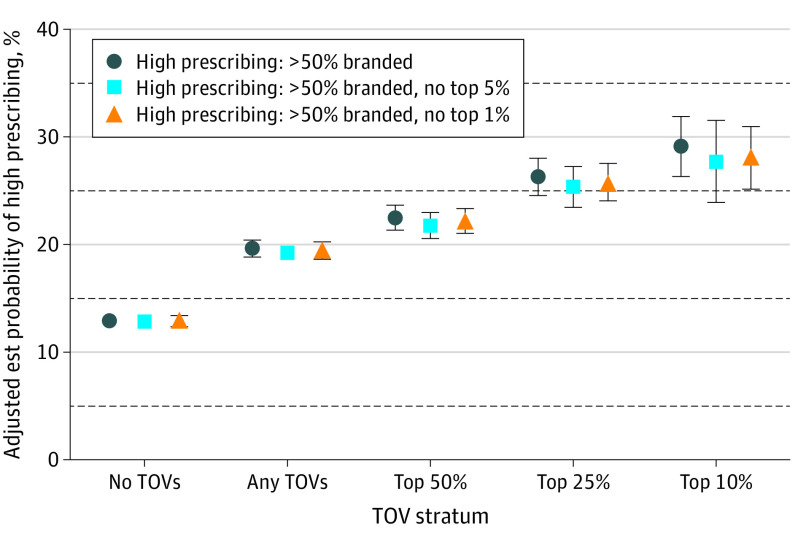
Association Between Industry-Reported Transfers of Value (TOV) and Branded Prostaglandin Analog Use Points show estimated probability of using more than 50% branded prostaglandin analogs with 95% CIs when comparing membership in each reported TOV stratum to nonmembership while controlling for other variables. Est indicates estimated.

The predicted probabilities of being a high prescriber are similar when excluding the top 5% of reported TOV recipients (more than $521 in 2018) and the top 1% of recipients (more than $9500 in 2018) from the model. This suggests that the association is not driven by a few prescribers with very high TOV (Figure 3). The magnitude of change in branded prescribing patterns was similar when defining high prescribers as more than 75% branded PGA use eTable 3 in the Supplement.

**Figure 4.**
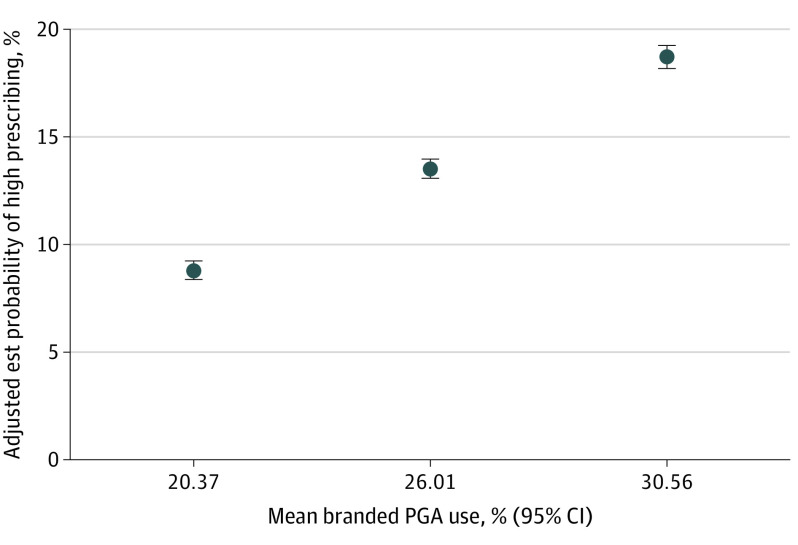
Association Between Local Area Preferences and Individual Prescribing Points show estimated marginal probabilities of using more than 50% branded prostaglandin analogs (PGAs) with 95% CIs at the quartiles of mean branded PGA use rates at the 3-digit zip code level. Est indicates estimated.

Prescriber sex (odds ratio [OR], 1.00 [95% CI, 1.60-1.87]), urban location (OR, 0.92 [95% CI, 0.83-1.01]), specialty (OR, 1.02 [95% CI, 0.93-1.11]), and glaucoma subspecialty (OR, 1.27 [95% CI, 1.043-1.55]) were not associated with high branded PGA use in the primary model (eTable 3 in the Supplement). Prescribers tended to follow the prescribing patterns of their peers in the same geographic location when comparing predicted probabilities of high prescribing at the quartiles of area PGA use rates, which were 8.8% (95% CI, 8.4%-9.2%; *P* < .001) for areas with 20% branded PGA use; 13.5% (95% CI, 13.1%-13.7%; *P* < .001) for areas with 26% use; and 18.7% (95% CI, 18.2%-19.2%; *P* < .001) for areas with 31% use (Figure 4). Total prescribing volume was inversely associated with being a high prescriber comparing predicted probabilities at the quartiles of total prescribing volume, which were 19.4% (95% CI, 18.5%-20.3%; *P* < .001) for 831 days supplied, 15.4% (95% CI, 15.0%-15.9%; *P* < .001) for 1778 days, and 11.6% (95% CI, 11.0%-12.2%; *P* < .001) for 4319 days (eFigure 3 in the Supplement).

Subgroup sensitivity analysis showed similar findings when the model was applied to optometrists and ophthalmologists separately (eTable 4 in the Supplement). Additional sensitivity analysis evaluating the association of reported receipt of more than the median TOV across separate TOV types (food, travel, speaking fees, consulting, and other) found food and speaking fees to have statistically significant associations with high prescribing (eTable 5 in the Supplement). These 2 categories accounted for more than 60% of total reported TOV, with the former being the most common form of reported TOV (eTable 6 in the Supplement).

## Discussion

Reported receipt of any nonresearch TOV from PGA manufacturers, regardless of amount, is associated with an almost doubled probability of prescribing branded PGAs. The median reported TOV was only $65.

These findings are consistent with research on industry TOV and anti–vascular endothelial growth factor agent prescribing.^13,14^ The important difference is that this study did not involve additional financial incentives posed by Part B buy and bill. These results are also consistent with findings in other medical specialties.^3,4,5,7,8,35,37,39^ Additionally, these results suggest that the association follows a dose response, with the likelihood of being a high prescriber of branded PGAs increasing from 13% to nearly 30% at the top decile of reported TOV recipients.

The previously documented independent associations between prescriber sex^5,13,34,36,37^ and urban location^5^ and branded drug use were not found when looking at PGAs. The finding relating 3-digit zip code–level branded PGA use to individual prescribing patterns is consistent with previous work showing that physicians tend to practice similarly within a locality.^44^ The inclusion of branded drug use at the local area level as a control variable is novel in this type of study, based on our literature review. It is possible that this control variable accounts for the lack of association seen with other control variables compared with prior studies with other drugs. Additionally, the inverse correlation between total prescribing volume and high branded drug use also differed from previous findings.^5,34^ This association might be attributable to greater experience with glaucoma management and understanding of determinants of treatment adherence or to differences in practice characteristics.

While we cannot determine the motivations of clinicians who frequently prescribe branded PGAs or the reason why individual patients were prescribed a branded PGA, reported receipt of industry TOV does seem to be an important factor associated with branded PGA use. The dose-response association suggests that the magnitude of TOV matters, but the relatively low value of typical reported TOV suggests that monetary gain is not the sole or even primary driver of this association. Assuming this is true, existing policies regarding disclosures of financial conflicts of interest above a certain magnitude would then be insufficient in limiting the industry influence.

We examined the literature on how small TOV might exert their effect. Work in social psychology provides some magnitude-independent explanations. These are the exposure effect, which creates a preference for something seen before; the frequency effect, which creates greater willingness to accept an idea (like 1 drug is superior to another) if repeatedly exposed to the idea; and implicit social pressure toward reciprocity.^45,46^ Reciprocity can certainly encompass the sense of collegiality that may emerge after repeated interactions and meals with sales representatives but can also be triggered by gifts and gestures between relative strangers. The susceptibility of health care professionals to these influences can then be heightened by motivated reasoning and illusory superiority—the impulse to believe that physicians are as impartial as they would like to be and that society expects them to be.^45^ This was shown in one study of 467 resident physicians wherein 61% believed they were unbiased by interactions with pharmaceutical companies but believed that the same was true for only 16% of their peers.^47^

These forces work at an unconscious level, demonstrated in a study that found physicians’ prescribing behavior changed in the months following industry-sponsored grand rounds, despite no longer remembering the sponsor,^48^ and another that found that prescribing behaviors changed after physicians attended an industry-sponsored continuing medical education event, despite being queried beforehand whether they believed the event would change their behavior.^49^ For these reasons, it has been suggested that the term *conflicts of interest* itself unhelpfully places undue emphasis on volitional decision-making and financial incentives. Instead, these processes could be considered part of human implicit social cognition,^45^ which cannot be curbed by mere awareness or force of will.^45,50^ So, while ophthalmologists and optometrists may feel that a meal or other small TOV may not influence their behavior, our data suggest that it might.

### Limitations

A limitation of this and other studies on this topic is the use of cross-sectional data, which makes it difficult to infer causality between reported industry TOV and branded drug use. It is possible that prescribers who prefer branded options become targeted for more attention from sales representatives, although this could still produce a reinforcing effect. The availability of generic bimatoprost starting in 2016 may be an additional confounder, but Medicare claims in 2018 for branded Lumigan (Allergan) still overshadowed claims for generic bimatoprost, which itself cost Medicare significantly more per unit than generic latanoprost.^51^

## Conclusions

There may be cases where industry interaction helps prescribers choose more effective agents for patients. However, with PGAs options are comparable,^22^ so the 2-fold increased likelihood of preferentially using branded PGAs associated with reported receipt of any TOV could pose a significant cost burden to patients over the course of their disease, which may reduce adherence to medical treatment regimens and worsen outcomes.^20,21^ Prospective, longitudinal investigation is needed to evaluate the potential of effects of industry TOV on glaucoma outcomes. Still, for optometrists and ophthalmologists who care for patients with glaucoma, the results of this study suggest a need to revisit policies and attitudes regarding industry interaction.

## Notes

Supplement. eFigure 1. Inclusion/Exclusion Criteria eFigure 2. Specialty Inference eFigure 3. Total PGA Prescribing and Branded PGA Use eTable 1. Simple Correlation of variables eTable 2. Reported Transfers of Value (TOVs) from Makers of PGAs in 2018, by Company eTable 3. Full Regression Outputs eTable 4. Sub-Group Analysis eTable 5. Reported Transfers of Value Type Analysis eTable 6. Reported Transfers of Value, by Type
