# Supplementary material for: Association Between Open Payments–Reported Industry Transfers of Value and Prostaglandin Analog Prescribing in the US
Source: JAMA Ophthalmol. 2022 Jul 28;140(9):855–62. doi: 10.1001/jamaophthalmol.2022.2757 (PMC9335252; doi:10.1001/jamaophthalmol.2022.2757)
Supplement: Supplement. — eFigure 1. Inclusion/Exclusion Criteria eFigure 2. Specialty Inference eFigure 3. Total PGA Prescribing and Branded PGA Use eTable 1. Simple Correlation of variables eTable 2. Reported Transfers of Value (TOVs) from Makers of PGAs in 2018, by Company eTable 3. Full Regression Outputs eTable 4. Sub-Group Analysis eTable 5. Reported Transfers of Value Type Analysis eTable 6. Reported Transfers of Value, by Type [file jamaophthalmol-e222757-s001.pdf]

Supplemental Online Content

Nguyen AM, Anderson KE, Anderson G, Johnson TV. Association between open payments-reported industry transfers of value and prostaglandin analog prescribing in the US. *JAMA Ophthalmol*. Published online July 28, 2022. doi:10.1001/jamaophthalmol.2022.2757

eFigure 1. Inclusion/Exclusion Criteria

eFigure 2. Specialty Inference

eTable 1. Simple Correlation of Variables

eTable 2. Reported Transfers of Value (TOVs) from Makers of PGAs in 2018, by Company

eTable 3. Full Regression Outputs

eFigure 3. Total PGA Prescribing and Branded PGA Use

eTable 4. Sub-Group Analysis

eTable 5. Reported Transfers of Value Type Analysis

eTable 6. Reported Transfers of Value, by Type

This supplemental material has been provided by the authors to give readers additional information about their work.

© 2022 Nguyen et al. *JAMA Ophthalmology.*

eFigure 1: Inclusion/Exclusion Criteria

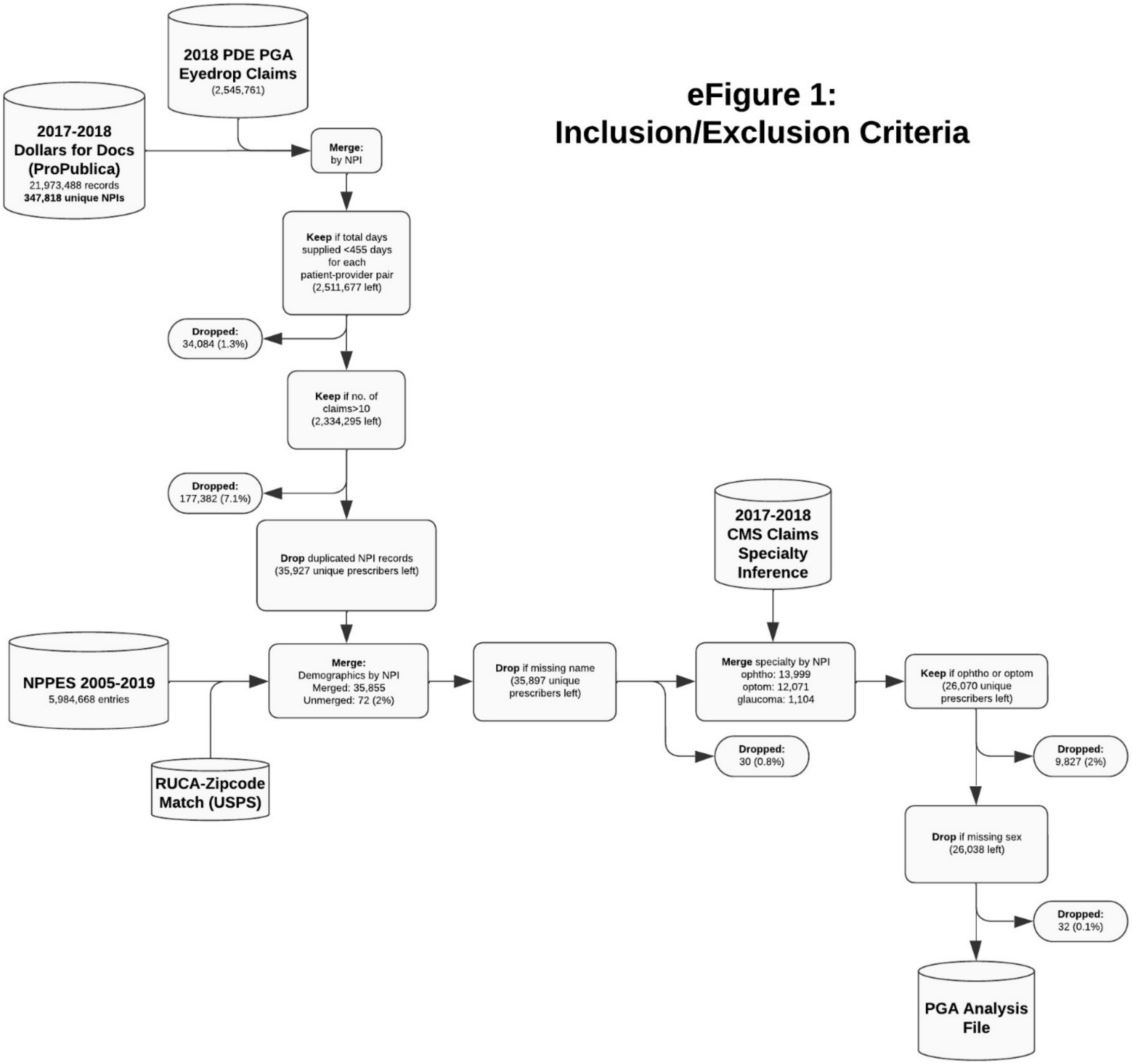

© 2022 Nguyen et al. *JAMA Ophthalmology.*

eFigure 2: Specialty Inference

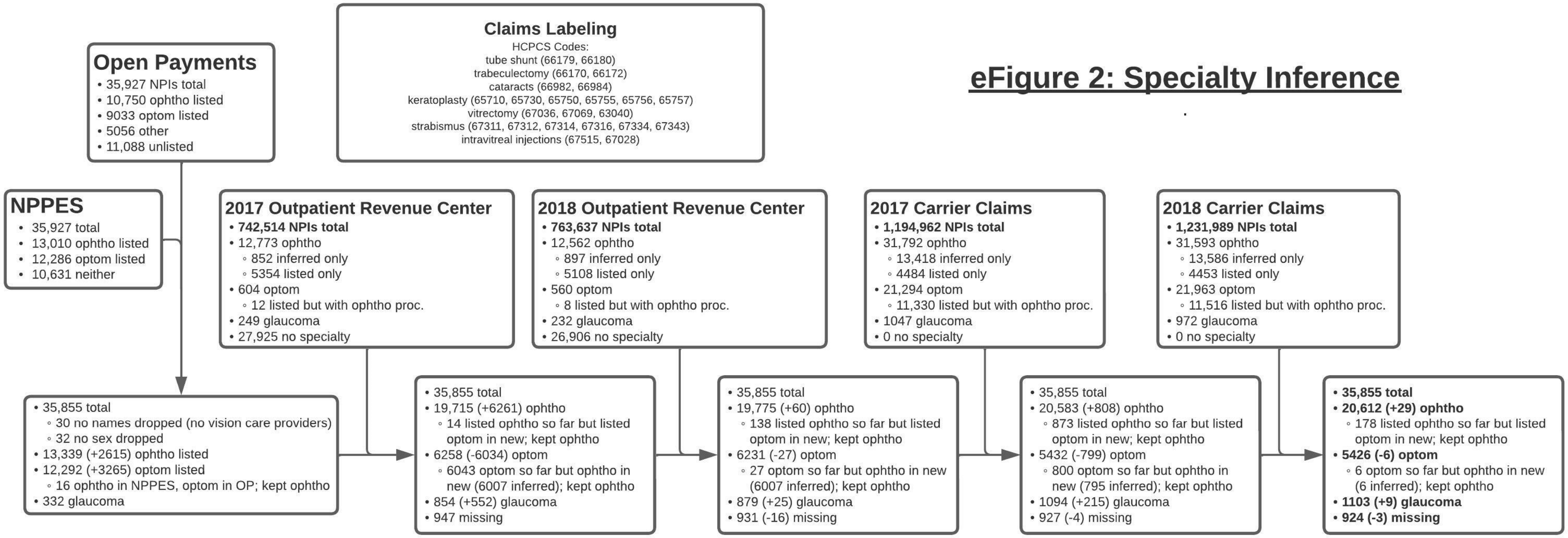

© 2022 Nguyen et al. *JAMA Ophthalmology.*

eTable 1: Simple Correlation of Variables

	Any Reported TOVs	Top 50% TOVs[1]	Top 25% TOVs[2]	Top 10% TOVs[3]	>50% branded PGA use	>75% branded PGA use	Male sex	Urban location	Ophthalmology[4]	Glaucoma	Total Prescribing Volume
Top 50% TOVs	0.6211	1.0000									
Top 25% TOVs	0.4161	0.6700	1.0000								
Top 10% TOVs	0.2553	0.4111	0.6136	1.0000							
>50% branded PGAs	0.0790	0.0871	0.0899	0.0711	1.0000						
>75% branded PGAs	0.0192	0.0241	0.0335	0.0301	0.4944	1.0000					
Male sex	0.0240	0.0117	0.0087	0.0229	-0.0155	-0.0133	1.0000				
Urban location	0.1842	0.1391	0.1151	0.0772	0.0074	0.0072	-0.0332	1.0000			
Ophthalmology	0.0707	0.0628	0.0510	0.0453	-0.0269	-0.0396	0.1510	-0.0321	1.0000		
Glaucoma	0.1297	0.1533	0.1664	0.1673	-0.0150	-0.0301	0.0045	0.0739	0.1079	1.0000	
Local Branded Use	0.1340	0.0967	0.0851	0.0594	0.2492	0.1581	-0.0096	0.0970	-0.0106	0.0039	1.0000
Total Prescribing Volume	0.2763	0.1987	0.1838	0.1378	-0.1099	-0.1500	0.1199	0.1108	0.2336	0.2930	0.0144

[1]>$65
[2]>$147
[3]>$288
[4]compared to optometrists

© 2022 Nguyen et al. *JAMA Ophthalmology.*

**eTable 2:** Reported Transfers of Value (TOVs) from Makers of PGAs in 2018, by Company			
	Median Dollars (IQR)—$	Median no. of TOVs (IQR)	Total Value (%)
**Overall**	65 (24 to 147)	2 (1 to 4)	5 060 346 (100%)
**Novartis**	26 (17 to 60)	1 (1 to 3)	577 128 (11%)
**Allergan**	63 (22 to 144)	2 (1 to 4)	4 445 047 (88%)
**Merck**	77 (26 to 156)	1 (1 to 2)	38 170 (1%)

© 2022 Nguyen et al. *JAMA Ophthalmology.*

eTable 3: Full Regression Outputs																
Main model, High Prescribing: >50%, regression output																
TOV Stratum	Any Reported TOV				Top 50%				Top 25%				Top 10%			
	N=26038		Psuedo-R^2= .0964		N=26038		Psuedo-R^2= .0973		N=26038		Psuedo-R^2= .0973		N=26038		Psuedo-R^2= .0933	
	**Odds Ratio**	**95% CI**		**P-value**	**Odds Ratio**	**95% CI**		**P-value**	**Odds Ratio**	**95% CI**		**P-value**	**Odds Ratio**	**95% CI**		**P-value**
TOV Stratum Membership	1.730	1.603	1.866	0.000	1.937	1.777	2.111	0.000	2.328	2.090	2.593	0.000	2.591	2.214	3.031	0.000
Male Sex	1.007	0.931	1.090	1.000	1.008	0.931	1.091	1.000	1.007	0.931	1.090	1.000	0.995	0.920	1.077	1.000
Urban Location	0.919	0.834	1.013	0.979	0.939	0.853	1.035	1.000	0.954	0.866	1.632	1.000	0.989	0.899	1.088	1.000
Ophthalmology	1.016	0.931	1.110	1.000	1.008	0.923	1.101	1.000	1.013	0.928	1.106	1.000	1.016	0.931	1.110	1.000
Glaucoma	1.270	1.043	1.547	0.187	1.177	0.966	1.435	1.000	1.177	0.934	1.392	1.000	1.155	0.945	1.411	1.000
Local Area Branded Use (%)	1.091	1.085	1.096	0.000	1.092	1.087	1.097	0.000	1.092	1.087	1.097	0.000	1.092	1.088	1.097	0.000
Total Prescribing Volume (log unit)	0.670	0.631	0.713	0.000	0.672	0.631	0.715	0.000	0.671	0.631	0.713	0.000	0.678	0.638	0.721	0.000
2-5 formularies	1.468	1.195	1.804	0.002	1.507	1.228	1.852	0.001	1.528	1.245	1.878	0.001	1.556	1.267	1.910	0.000
5-10 formularies	1.385	1.108	1.723	0.044	1.464	1.175	1.824	0.007	1.507	1.209	1.876	0.003	1.550	1.245	1.929	0.001
>10 formularies	1.406	1.090	1.813	0.095	1.528	1.185	1.968	0.012	1.571	1.219	2.024	0.005	1.645	1.278	2.117	0.001

*Pseudo-R^2 is a goodness-of-fit metric for comparing iterations of logistic regressions analogous to R^2 values in ordinary least squares (OLS) linear regression
**P-values adjusted by Bonferroni correction

High Prescribing: >50% Branded Use, Excluding top 5%, regression output																
TOV Stratum	Any Reported TOV				Top 50%				Top 25%				Top 10%			
	N=25553		Psuedo-R^2= .0987		N=25553		Psuedo-R^2= .0987		N=25553		Psuedo-R^2= .0979		N=25553		Psuedo-R^2= .0933	
	**Odds Ratio**	**95% CI**		**P-value**	**Odds Ratio**	**95% CI**		**P-value**	**Odds Ratio**	**95% CI**		**P-value**	**Odds Ratio**	**95% CI**		**P-value**
TOV Stratum Membership	1.687	1.560	1.824	0.000	1.852	1.692	2.024	0.000	2.206	1.956	2.484	0.000	2.396	1.929	2.980	0.000
Male Sex	0.999	0.922	1.082	1.000	1.001	0.925	1.084	1.000	1.002	0.926	2.270	1.000	0.993	0.917	1.075	1.000
Urban Location	0.912	0.827	1.005	0.695	0.936	0.850	1.030	1.000	0.953	0.866	1.050	1.000	0.989	0.899	1.089	1.000
Ophthalmology	1.012	0.926	1.106	1.000	1.006	0.920	1.099	1.000	1.011	0.926	1.105	1.000	1.014	0.929	1.108	1.000
Glaucoma	1.111	0.890	1.385	1.000	1.050	0.841	1.311	1.000	1.044	0.834	1.305	1.000	1.091	0.874	1.363	1.000
Local Area Branded Use (%)	1.091	1.087	1.096	0.000	1.092	1.088	1.097	0.000	1.092	1.088	1.097	0.000	2.435	1.088	1.099	0.000
Total Prescribing Volume (log unit)	0.647	0.608	0.690	0.000	0.651	0.610	0.693	0.000	0.651	0.611	0.694	0.000	0.000	0.619	0.703	0.000
2-5 formularies	1.484	1.208	1.824	0.002	1.525	1.241	1.874	0.001	1.548	1.260	2.568	0.000	1.575	1.283	1.935	0.000
5-10 formularies	1.432	1.147	1.788	0.017	1.516	1.215	1.893	0.003	1.557	1.249	1.944	0.001	1.597	1.281	1.992	0.000
>10 formularies	1.470	1.135	1.902	0.038	1.598	1.236	2.067	0.004	1.644	1.271	2.123	0.002	1.718	1.330	2.217	0.000

*Pseudo-R^2 is a goodness-of-fit metric for comparing iterations of logistic regressions analogous to R^2 values in ordinary least squares (OLS) linear regression
**P-values adjusted by Bonferroni correction

© 2022 Nguyen et al. *JAMA Ophthalmology.*

High Prescribing: >50% Branded Use, excluding top 1%, regression output																
TOV Stratum	Any Reported TOV				Top 50%				Top 25%				Top 10%			
	N=25941		Psuedo-R^2= .0965		N=25941		Psuedo-R^2= .0971		N=25941		Psuedo-R^2= .0925		N=25941		Psuedo-R^2= .0925	
	**Odds Ratio**	**95% CI**		**P-value**	**Odds Ratio**	**95% CI**		**P-value**	**Odds Ratio**	**95% CI**		**P-value**	**Odds Ratio**	**95% CI**		**P-value**
TOV Stratum Membership	1.713	1.586	1.850	0.000	1.900	1.744	2.073	0.000	2.442	2.071	2.883	0.000	2.442	2.071	2.883	0.000
Male Sex	1.004	0.928	1.087	1.000	1.006	0.930	1.088	1.000	0.995	0.923	1.076	1.000	0.995	0.919	1.076	1.000
Urban Location	0.919	0.834	1.012	0.931	0.939	0.853	1.035	1.000	0.990	0.899	1.090	1.000	0.990	0.899	1.090	1.000
Ophthalmology	1.017	0.931	1.111	1.000	1.010	0.454	1.103	1.000	1.197	0.932	1.112	1.000	1.018	0.932	1.112	1.000
Glaucoma	1.229	1.003	1.504	0.516	1.147	0.935	1.406	1.000	1.149	0.935	1.411	1.000	1.149	0.935	1.411	1.000
Local Area Branded Use (%)	1.091	1.085	1.096	0.000	1.092	1.087	1.097	0.000	1.092	1.035	1.097	0.000	1.092	1.088	1.097	0.000
Total Prescribing Volume (log unit)	0.666	0.626	0.708	0.000	0.667	0.627	0.710	0.000	0.676	0.634	0.717	0.000	0.674	0.634	0.717	0.000
2-5 formularies	1.468	1.195	1.804	0.003	1.507	1.228	1.852	0.001	1.556	1.269	2.578	0.001	1.556	1.269	1.910	0.000
5-10 formularies	1.399	1.122	1.744	0.032	1.481	1.189	1.846	0.001	1.525	1.224	1.898	0.002	1.565	1.257	1.950	0.001
>10 formularies	1.416	1.097	1.829	0.082	1.540	1.194	1.986	0.010	1.584	1.229	2.042	0.004	1.659	1.287	2.136	0.001

*Pseudo-R^2 is a goodness-of-fit metric for comparing iterations of logistic regressions analogous to R^2 values in ordinary least squares (OLS) linear regression

**P-values adjusted by Bonferroni correction

High prescribing: >75% Branded Use, regression output																
TOV Stratum	Any Reported TOV				Top 50%				Top 25%				Top 10%			
	N=26038		Psuedo-R^2= .1472		N=26038		Psuedo-R^2= .1461		N=26038		Psuedo-R^2= .1479		N=26038		Psuedo-R^2= .1456	
	**Odds Ratio**	**95% CI**		**P-value**	**Odds Ratio**	**95% CI**		**P-value**	**Odds Ratio**	**95% CI**		**P-value**	**Odds Ratio**	**95% CI**		**P-value**
TOV Stratum Membership	1.749	1.586	2.002	0.000	1.833	1.744	2.073	0.000	2.418	1.996	2.883	0.000	2.939	2.239	3.857	0.000
Male Sex	1.093	0.928	1.255	1.000	1.090	0.930	1.088	1.000	1.092	0.951	1.076	1.000	1.080	0.942	1.240	1.000
Urban Location	1.093	0.834	1.301	1.000	1.127	0.853	1.035	1.000	1.130	0.950	1.090	1.000	1.170	0.985	1.391	0.807
Ophthalmology	1.041	0.931	1.204	1.000	1.037	0.454	1.103	1.000	1.036	0.895	1.112	1.000	1.036	0.895	1.198	1.000
Glaucoma	1.091	1.003	1.852	1.000	1.020	0.935	1.406	1.000	0.979	0.569	1.411	1.000	0.973	0.564	1.679	1.000
Local Area Branded Use (%)	1.094	1.085	1.102	0.000	1.095	1.087	1.097	0.000	1.095	1.088	1.097	0.000	1.095	1.088	1.103	0.000
Total Prescribing Volume (log unit)	0.480	0.626	0.528	0.000	0.475	0.627	0.710	0.000	0.476	0.424	0.717	0.000	0.477	0.425	0.535	0.000
2-5 formularies	0.621	0.488	0.791	0.000	0.640	1.228	1.852	0.003	0.643	0.506	2.578	0.004	0.658	0.518	0.837	0.007
5-10 formularies	0.452	0.335	0.611	0.000	0.480	1.154	1.846	0.000	0.483	0.359	1.950	0.000	0.503	0.373	0.676	0.000
>10 formularies	0.393	0.261	0.592	0.000	0.428	1.194	1.986	0.000	0.424	0.281	2.136	0.000	0.448	0.298	0.673	0.001

*Pseudo-R^2 is a goodness-of-fit metric for comparing iterations of logistic regressions analogous to R^2 values in ordinary least squares (OLS) linear regression

**P-values adjusted by Bonferroni correction

© 2022 Nguyen et al. *JAMA Ophthalmology.*

eFigure 3: Total PGA Prescribing and Branded PGA Use

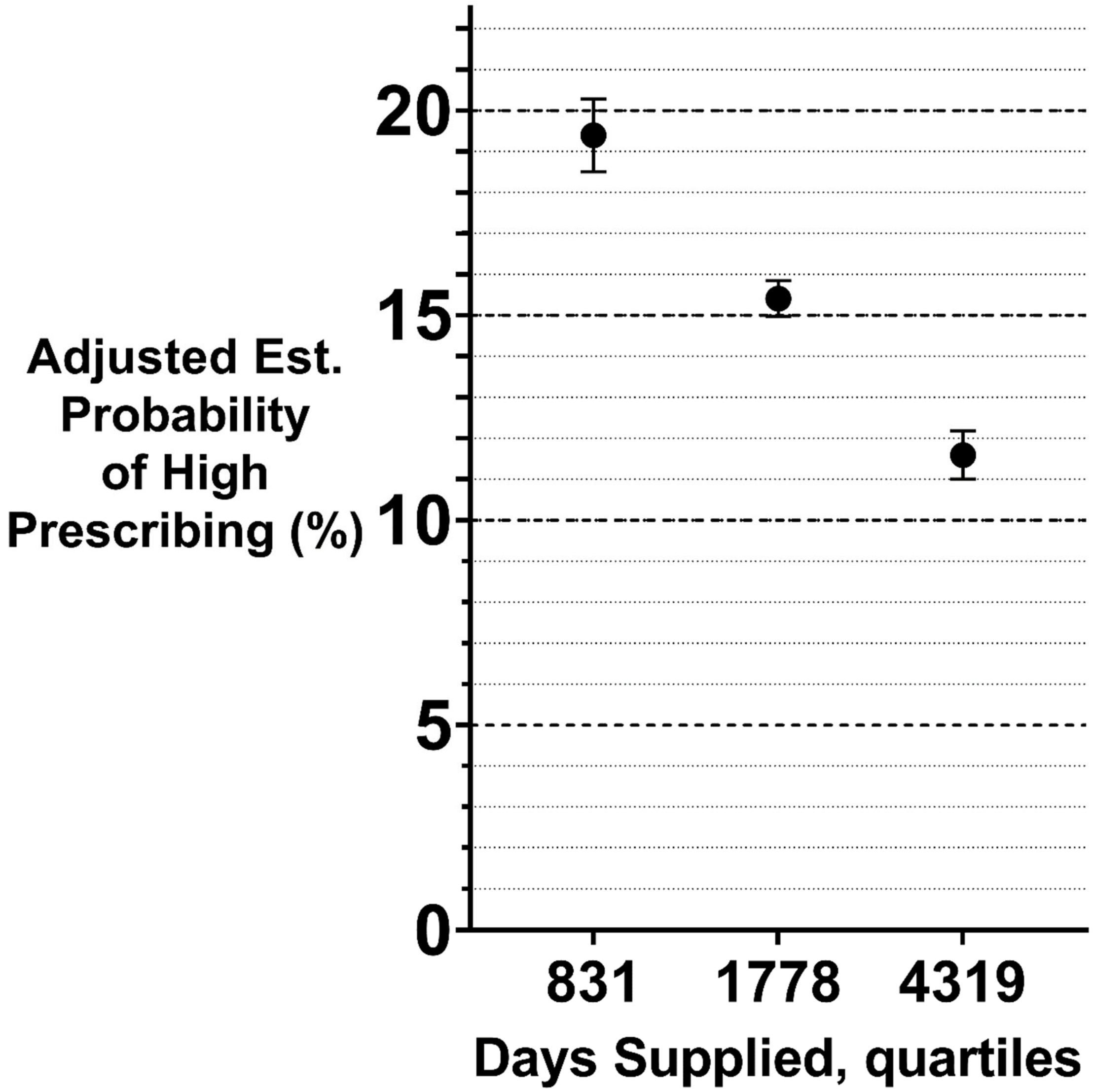

© 2022 Nguyen et al. *JAMA Ophthalmology.*

eTable 4: Sub-Group Analysis								
	**Optometrists**				**Ophthalmologists**			
	N=5426		Psuedo-R^2= .1249		N=26038		Psuedo-R^2= .1532	
	**Odds Ratio**	**95% CI**		**P-value**	**Odds Ratio**	**95% CI**		**P-value**
**Any reported transfer of value**	1.581	1.344	1.859	0.000	1.777	1.629	1.927	0.000
**Top 50%**	1.970	1.629	2.382	0.000	1.939	1.761	2.134	0.000
**Top 25%**	2.081	1.626	2.664	0.000	2.387	2.117	2.691	0.000
**Top 10%**	2.421	1.677	3.494	0.000	2.646	2.226	3.146	0.000
**Male Sex**	1.112	0.956	1.293	1.000	0.969	0.883	1.062	1.000
**Urban Location**	0.925	0.744	1.150	1.000	0.919	0.824	1.024	1.000
**Glaucoma**	N/A	N/A	N/A	N/A	1.264	1.036	1.542	0.231
**Local Area Branded Use (%)**	1.085	1.075	1.095	0.000	1.093	1.088	1.099	0.000
**Total Prescribing Volume (log unit)**	0.633	0.552	0.725	0.000	0.678	0.633	0.726	0.000
	Top 50%: >$53.72<br>Top 25%: >$131.03<br>Top 10%: >$243.25				Top 50%: >$67.86<br>Top 25%: >$149.53<br>Top 10%: >$317.02			

*Pseudo-R^2 is a goodness-of-fit metric for comparing iterations of logistic regressions analogous to R^2 values in ordinary least squares (OLS) linear regression
**P-values adjusted by Bonferroni correction

© 2022 Nguyen et al. *JAMA Ophthalmology.*

eTable 5: Reported Transfer of Value Type Analysis				
	N=5426		Psuedo-R^2= .1249	
	**Odds Ratio**	**95% CI**		**P-value**
**Food***	1.925	1.763	2.100	0.000
**Travel***	1.230	0.680	2.226	1.000
**Speaking***	2.563	1.406	4.669	0.034
**Consulting***	0.912	0.544	1.530	1.000
**Other***	0.756	0.308	1.855	1.000
**Male Sex**	1.005	0.929	1.088	1.000
**Urban Location**	0.938	0.852	1.034	1.000
**Ophthalmology**	1.008	0.923	1.105	1.000
**Glaucoma**	1.142	0.934	1.397	1.000
**Local Area Branded Use (%)**	1.092	1.087	1.097	0.000
**Total Prescribing Volume (log units)**	0.669	0.629	0.712	0.000

*Analysis performed as single model with dummy variables for receiving more than the median total payments amount for each payment type

**Pseudo-R^2 is a goodness-of-fit metric for comparing iterations of logistic regressions analogous to R^2 values in ordinary least squares (OLS) linear regression

***P-values adjusted by Bonferroni correction

© 2022 Nguyen et al. *JAMA Ophthalmology.*

eTable 6: Reported Transfers of Value, by Type		
**Type**	**No. of Prescribers Receiving in 2018**	**Total Value**
Food	9642	$1,193,544
Travel	266	$596,998
Speaking	191	$1,976,413
Consulting	280	$1,282,281
Other	101	$8375

eFigure 1
Inclusion/Exclusion Criteria. Flowchart shows process for merging different data sets and the inclusion/exclusion criteria applied.

eFigure 2
Specialty Inference. Flowchart shows process for inferring prescriber specialty from Carrier and Outpatient Claims. In discrepancies, designation as an ophthalmologist inferred from procedures billed was allowed to override listed specialty, which occurred for about half of providers listed as “optometrists.” Because of this, discrepancies in listed specialty between different datasets were retained as ophthalmologists.

eFigure 3
Association between Total PGA Prescribing and Branded PGA Use. Points show estimated marginal probabilities of using more than 50% branded PGAs with 95% confidence intervals at the quartiles of total PGA prescribing, measured in total days supplied of PGA eyedrops.

© 2022 Nguyen et al. *JAMA Ophthalmology.*
